# Guidelines for oral fluid-based surveillance of viral pathogens in swine

**DOI:** 10.1186/s40813-020-00168-w

**Published:** 2020-10-19

**Authors:** Alexandra Henao-Diaz, Luis Giménez-Lirola, David H. Baum, Jeffrey Zimmerman

**Affiliations:** Department of Veterinary Diagnostic and Production Animal Medicine, College of Veterinary Medicine, Veterinary Medical Research Institute, Iowa State University, Ames, Iowa 50011 USA

**Keywords:** Oral fluids, Surveillance, Viral diseases, ELISA, RT-PCR

## Abstract

Recent decades have seen both rapid growth and extensive consolidation in swine production. As a collateral effect, these changes have exacerbated the circulation of viruses and challenged our ability to prevent, control, and/or eliminate impactful swine diseases. Recent pandemic events in human and animal health, e.g., SARS-CoV-2 and African swine fever virus, highlight the fact that clinical observations are too slow and inaccurate to form the basis for effective health management decisions: systematic processes that provide timely, reliable data are required. Oral fluid-based surveillance reflects the adaptation of conventional testing methods to an alternative diagnostic specimen. The routine use of oral fluids in commercial farms for PRRSV and PCV2 surveillance was first proposed in 2008 as an efficient and practical improvement on individual pig sampling. Subsequent research expanded on this initial report to include the detection of ≥23 swine viral pathogens and the implementation of oral fluid-based surveillance in large swine populations (> 12,000 pigs). Herein we compile the current information regarding oral fluid collection methods, testing, and surveillance applications in swine production.

## Background

Between 1968 and 2018, the worldwide swine inventory increased from 550 to 981 million pigs (+ 78%), with the most marked growth in the developing regions of the world, i.e., Africa + 504%, Asia + 137%, and South America + 59% [1]. Over the same period, albeit with regional variations, the majority of pig production moved from smaller, farrow-to-finish enterprises into larger, multi-site production systems that are highly dependent upon the interchange of animals, people, equipment, and sundries between production sites; a process that connects farms and moves infectious agents between them [2, 3]. From an animal health perspective, larger pig populations combined with extensive interactions between production sites produce conditions that promote the circulation of infectious diseases within/between farms while simultaneously challenging our ability to control them [4, 5]. From a business perspective, these changes have limited our ability to avoid or control the economic impact of adverse disease events and, thus, place producers at greater financial risk [6].

In 1982, Calvin Schwabe, responding to the emerging problem of multi-factorial “production diseases”, noted that the simple causal models described by Koch and Pasteur no longer applied, i.e., predisposing causes, latency, carriers, opportunistic pathogens [4, 5]. To adapt to these new conditions, he recommended on-going surveillance to establish baseline levels of disease “against which effects of intervention (control) efforts can be measured”. No one followed Schwabe’s advice - perhaps because the serum-based surveillance methods of the time were too cumbersome and expensive for routine use in commercial swine herds.

Since the publication of Schwabe’s comments, a variety of diagnostic alternatives to serum have been described, e.g., tonsil scrapings [7], tonsil biopsies [8], tonsil swabs [9], blood swabs [10], nasal swabs [11], nasal wash [12], buccal swabs [13], probang samples [14], and oral fluids [15]. These specimens offer new possibilities for surveillance, but are they diagnostically equivalent? Direct comparisons are rare in the published literature, but it is broadly recognized that the concentration of diagnostic targets changes in various diagnostic specimens over the course of an infection with corresponding specimen-dependent changes in test performance. Thurmond (2003), described this process as “disease transition stages”, and the corresponding changes in diagnostic performance for specific combinations of specimen and test as “diagnostic transition stages”. Thus, Henao-Diaz et al. (2020) showed that the probability of detecting porcine reproductive and respiratory syndrome virus (PRRSV) ribonucleic acid (RNA) in serum by reverse transcription polymerase chain reaction (RT-PCR) at 98 days post infection (DPI) was ~ 2% versus ~ 30% in lymphoid tissues (tonsil) by bioassay [16]. A complete discussion of the diagnostic performance issues intrinsic to various specimen is beyond the scope of this review, but these differences must be considered in designing sampling/testing protocols. With this caution in mind, the objective of this review is to present the fundamental concepts of oral fluid-based diagnostics and recommendations for their use in the field.

### Definitions

Saliva, mixed saliva, and oral fluid differ by composition and the method of collection [17]. *Saliva* is primarily produced by three pairs of salivary glands (parotid, submandibular, sublingual) and is collected using specific techniques, including cannulation of the salivary ducts. Parotid glands produce a serous secretion; submandibular and sublingual glands produce seromucous secretions rich in proteins and other serum-derived components [18, 19]. *Mixed or whole saliva*, the fluid collected from the buccal cavity by spitting or drainage, is a mixture of saliva and other constituents, i.e., mucosa transudate, gingival crevicular exudate, cell detritus, tracheal-nasal secretions, food debris, gastrointestinal reflux, and serum-derived compounds [18, 19]. *Oral fluid*, as defined by Atkinson et al. (1993), is the liquid collected by placing an absorptive device in the buccal cavity. Various commercial devices are available for collecting oral fluids from humans, but oral fluids are usually collected either from one individual pig or a group of pigs by suspending a length of cotton rope in the pen, then recovering the individual fluid or the aggregate fluid by compressing the rope [20].

### Oral fluids

As reviewed by Prickett and Zimmerman (2010), research on immunologic and diagnostic aspects of oral fluids from humans and domestic animals began roughly a century ago and has included work on some of the most impactful diseases of humans and livestock, e.g., poliovirus and food-and-mouth disease virus (FMDV). A key event in this timeline was the recognition that both human immunodeficiency virus (HIV) and HIV antibody (1986) were present in oral fluids from infected individuals; a finding that led to the development of commercial oral fluid HIV antibody tests (1995) [21]. For both humans and domestic animals, there are two major issues for oral fluid diagnostic assays: (1) antibody assays must account for the lower antibody concentration in oral fluid versus serum [22], and (2) PCR assays must account for the unique characteristics of the oral fluid matrix. In sum, diagnostic assays are not directly interchangeable between specimens and must be adapted to the oral fluid matrix to provide the best diagnostic performance [23, 24].

Oral fluid is a dynamic and complex matrix consisting of water, hormones, metabolites, electrolytes, enzymes, antibodies, mucins, and an assortment of other proteins [18, 25]. Oral fluid necessarily contains components produced in buccal-associated tissues, e.g., saliva and antibodies produced by plasma cells located in salivary glands and tonsils [26]. In addition, the oral cavity is covered with a protective layer of semipermeable mucosa tightly bound to the underlying connective tissue. The semipermeability of these tissues facilitates a continuous exchange between the circulatory/lymphatic systems and the buccal cavity by both passive and active processes: 1) ultrafiltration of small molecules through intercellular gap junctions, e.g., water, ions, hormones, urea; 2) transudation of components from capillaries associated with the mucosa; and 3) selective and/or receptor-mediated transport of larger molecules and lipophilic compounds from capillaries, e.g., antibodies, hormones, and other proteins. Likewise, a dynamic exchange of inorganic components, e.g., bicarbonate, chlorine, potassium, and phosphate, occurs as fluid moves through the salivary ducts, ultimately affecting the pH and tonicity of buccal fluids [19, 27].

In addition to physiologically intrinsic constituents, oral fluids usually contain enteric micro-organisms, feed components, drug compounds and metabolites recovered by the pigs from their environment. That is, as a result of pigs’ normal exploratory behavior, i.e., smelling, tasting, biting, and rooting, environmental diagnostic targets are collected in the buccal cavity [28] - some of which are subsequently passed onto the rope and into the oral fluid specimen. This is not a negative attribute of the specimen; to the contrary, the presence of such material broadens the diagnostic utility of the oral fluid sample [29, 30].

## Diagnostic potential of oral fluid specimens

### Detection of antibody in oral fluids

**Fig. 1:**
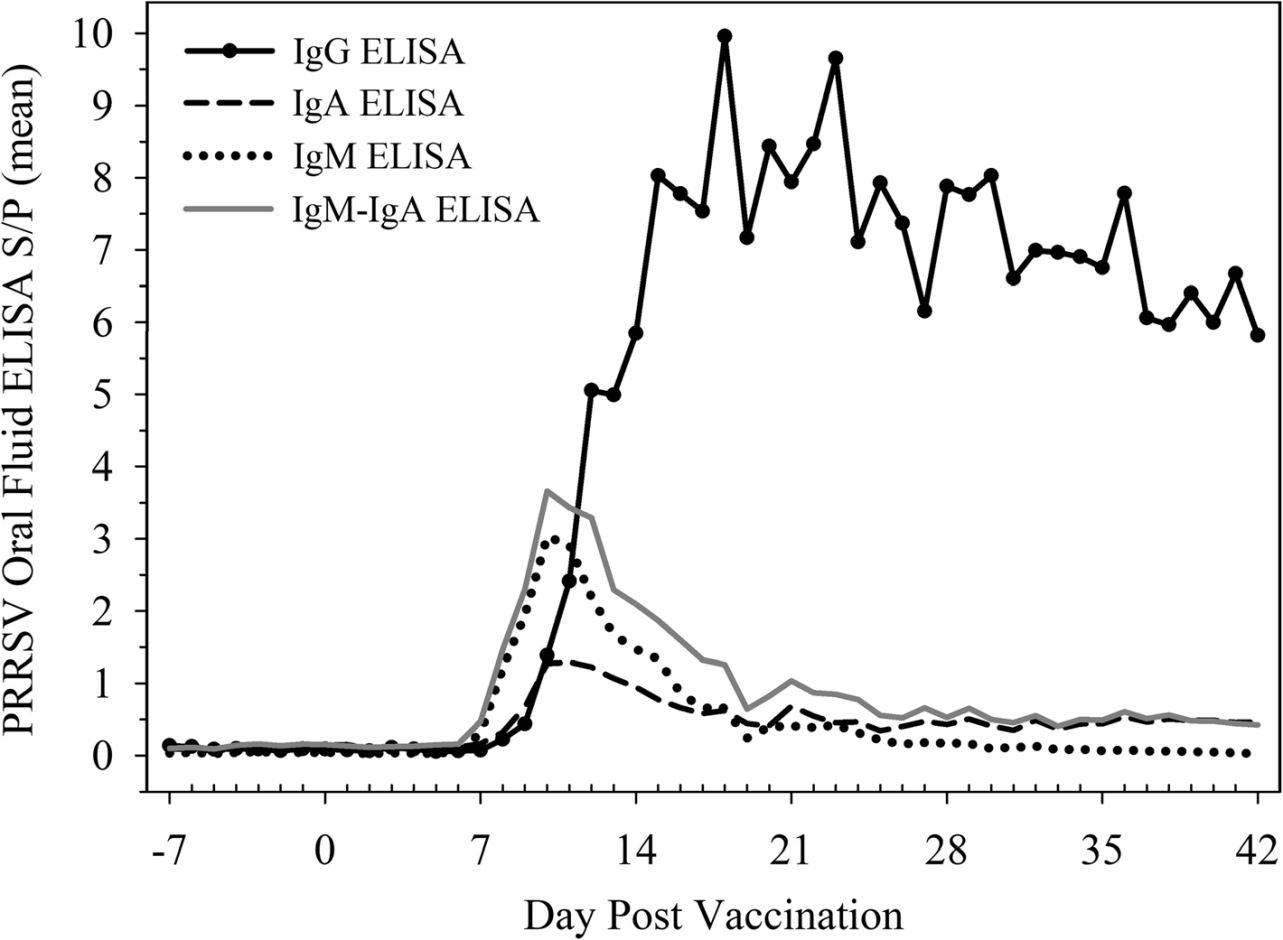
PRRSV antibody kinetics in oral fluid samples collected from 12 pigs vaccinated with a modified-live virus vaccine over the course of 50 days (− 7 to 42 DPV). Reprinted from Rotolo et al. (2018) [40], Veterinary Microbiology 214, 13–20 (copyright 2017) with permission from Elsevier (4812031256013)

Immunoglobulins (Ig) M, IgA, and IgG reach the buccal cavity by passive diffusion and/or via receptor-mediated transportation from the circulatory and/or lymphatic systems [31, 32]. In addition, all classes of antibody, including secretory IgA, are produced by plasma cells located in tissues associated with the buccal cavity [19, 23]. Serum and oral fluid antibody isotype kinetics are similar, as demonstrated in studies on the antibody responses to African swine fever virus (ASFV) [33], classical swine fever virus (CSFV) [34], influenza A virus (IAV) [35], porcine circovirus type 2 (PCV2) [36], and PRRSV [37]. As observed in serum, IgM is detected in oral fluids before IgA and IgG, but has a shorter half-life than other isotypes. IgA (primarily secretory IgA) appears earlier than IgG but usually IgG is the preferred target of oral fluid enzyme-linked immunosorbent assays (ELISAs) because its longer half-life and because usually provides for more diagnostically sensitive and specific assays [38, 39] (Fig. 1). Importantly, the isotype-specific responses against each pathogen must be investigated during the process of assay development because there are exceptions to this general rule. Thus, Bjustrom-Kraft et al. (2016) found that a porcine epidemic diarrhea virus (PEDV) oral fluid ELISA based on IgA detection provided the best diagnostic performance [41] and Rotolo et al. (2018a) demonstrated that an IgM-IgA PRRSV oral fluid ELISA detected infection in young pigs, even in the presence of circulating maternal IgG PRRSV antibody [40] (Fig. 1).

### Detection of nucleic acids in oral fluids

PCR (or RT-PCR, depending on the genetic composition of the pathogen considered) is a general approach for detecting nucleic acids that has been adapted to a variety of diagnostic specimens, including oral fluids (Table 1). Most typically, the detection of viral nucleic acids in oral fluids is coincident with viremia or viral replication in buccal tissues and/or the upper respiratory tract, but viruses in the environment will also be detected. For example, PEDV, porcine coronavirus (PCV2), and porcine delta coronavirus (PDCoV) shed in feces were likewise detected in oral fluids [41, 51, 54].

**Table 1:** Swine virus-specific nucleic acid and antibody detection reported in oral fluids^a^

Pathogen^b^	Nucleic acid detection	Antibody detection
APPV	Schwarz et al., 2017 [42]	not reported
ASFV	Grau et al., 2015 [43]	Giménez-Lirola et al., 2016 [33]
CSFV	Dietze et al., 2017 [44]	Panyasing et al., 2018c [34]
FMDV	Senthilkumaran et al., 2017b [45]	Poonsuk et al., 2018 [46]
HEV	Plut et al., 2020 [47]	not reported
IAV	Goodell et al., 2013 [11]	Panyasing et al., 2014 [48]
JEV	Lyons et al., 2018 [49]	not reported
NIPAH virus	Kasloff et al., 2019 [50]	not reported
PCV2	Wonziak et al., 2019 [51]	Prickett et al., 2011 [36]
PCV3	Guo et al., 2019 [52]	Bai et al., 2020 [53]
PDCoV	Homwong et al., 2016 [54]	not reported
PEDV	Bjustrom-Kraft et al., 2016 [41]	Bjustrom-Kraft et al., 2016 [41]
PHEV	Mora-Díaz et al., 2019 [55]	Mora-Díaz et al., 2019 [55]
PKV	Gauger et al., 2020 [56]	not reported
PPIV1	Park et al., 2019 [57]	not reported
PPV	Milek et al., 2019 [58]	not reported
PRCV	Magtoto et al., 2019 [59]	Magtoto et al., 2019 [59]
PRRSV	Decorte et al., 2015 [60]	Henao-Diaz et al., 2019 [38]
PRV	Panyasing et al., 2018a [61]	Panyasing et al., 2018a [61]
SVA	Hole et al., 2019 [62]	Hole et al., 2019 [62]
SVDV	Senthilkumaran et al., 2017a [63]	Senthilkumaran et al., 2017a [63]
TGEV	Magtoto et al., 2019 [59]	Magtoto et al., 2019 [59]
TTV	Ramirez et al., 2012 [64]	not reported

^a^ Table 1 provides examples of the detection of virus-specific nucleic acids and/or antibody in swine oral fluids, i.e., is not comprehensive

^b^ Acronyms defined in the list of abbreviations and terms

PCR is considered the best method for “early” detection of viral infections in oral fluids, but “early” varies among pathogens. For example, FMDV replicates in the soft palate, pharyngeal epithelium, and tonsils, and may be detected in oral fluids at ≥1 DPE [65]. Pseudorabies virus (PRV) first replicates in epithelial cells of the upper respiratory tract and was detected in oral fluids at ≥3 DPE [61]. CSFV replicates in tonsils and lymphoid nodes has been detect in oral fluids at ≥5 DPE [66]. A further complication, is the variation in detection by PCR observed among strains of the same virus and even among specimens from the same animals. Weesendorp et al. (2009) described marked differences in detection among 3 strains of CSFV in a comparison of 8 different specimen types; feces, serum, and buccal specimens among them [67]. Similarly, Pepin et al., (2015) reported both isolate - and specimen-dependent differences in PRRSV detection in boars inoculated under experimental conditions [10] (Table 2).

**Table 2:** Early detection of PRRSV by PCR as a function of specimen and day post inoculation^a^

Specimen	Positivity (%) by day post inoculation						
	1	2	3	4	5	6	7
Serum	36.5	79.1	89.5	93.8	95.2	97.4	99.9
Blood swab	30.3	73.3	79.4	86.7	87.9	99.9	99.9
Oral fluid	3.6	59.0	89.4	97.6	99.9	99.9	99.9

^a^ Probability calculated using a binomial logistic regression model with estimates obtained using the least square methods. Reprinted from Pepin et al. (2015) Transboundary and Emerging Diseases 62, 295–304 (copyright 2013) with permission from John Wiley and Sons (4816070894659)

### Detection of infectious virus in oral fluids

With mixed success, some of the key viral pathogens of swine (FMDV, SVDV, IAV, SVA, and PRRSV) have been isolated from oral fluids under research conditions [7, 24, 45, 62, 63]. Virus isolation is not routinely used in surveillance and will not be addressed in this review. If virus recovery is an objective, it should be attempted on optimally-collected specimens from clinically-affected individuals.

### Similar … but not the same

Specimens that seem similar to oral fluids may have very different diagnostic characteristics. For example, Pepin et al. (2015a) compared testing results among oral fluids and “frothy saliva”, i.e., the buccal foam produced mature boars, in 15 boars for 14 days following administration of a commercial modified-live virus PRRSV vaccine. Between 1 to 14 days post vaccination (DPV), 71% (50/70) of oral fluids and 19% (13/70) of frothy saliva samples were positive for PRRSV RNA. Between 8 to 14 DPV, PRRSV ELISA antibody positivity rates were 69% (24/35) for oral fluids and 0% (0/35) for frothy saliva [10].

Similarly, differences in detection rates have been reported for pen-based oral fluids versus individual pig buccal or nasal swabs for animals inoculated with FMDV, IAV, Senecavirus A (SVA), and swine vesicular disease virus (SVDV) [45, 62, 63, 68]. In each study, oral fluids provided higher RNA detection rates than swabs (Table 3). These results may be explained by the fact that swabs inherently collect a smaller amount of target and typical collection procedures further dilute the sample. In particular, placing the swab in 1–2 ml of medium for transport to the laboratory reduces the concentration of the target in the sample and, therefore, the probability of detection [68]. In the case of buccal swabs, physiology also comes in to play. Salivary glands, ducts, and small vessels are innervated by sympathetic (fight-or-flight) and parasympathetic systems. The process of restraining a pig to collect the buccal swab induces a stress response that includes vasoconstriction of vessels supplying the buccal mucosa, salivary glands, and salivary ducts. This response reduces the flow of fluids to the mouth (“dry mouth”) and alters buccal fluid composition [19, 26, 27].

**Table 3:** Difference on nucleic detection rates in oral fluids and individual buccal or nasal swabs

Virus	Specimen	Rate of detection (%) by Day post inoculation (DPI)									
		1	2	3	4	5	6	8	10	12	15
FMD^a^	OF	75	100	100	100	100	75	*	*	100	*
	Swabs	4	33	61	100	89	50	*	*	25	*
IAV^b^	OF	100	100	75	25	38	29	29	17	0	0
	Swabs	100	100	100	75	13	0	0	0	0	0
PRRSV^c^	OF	0	20	60	80	100	100	100	80	100	20
	Swabs	0	0	0	40	20	60	0	20	20	20
SVA^d^	OF	*	67	*	100	*	100	100	100	*	100
	Swabs	*	92	*	100	*	75	67	75	*	8

* No data reported at the DPI or one day before or after the DPI

^a^ Mean of positivity rate estimated on pen-based oral fluids and individual buccal swabs collected from 24 pigs divided in 4 groups [45]

^b^ Mean of positivity rate estimated on pen-based oral fluids and individual nasal swabs collected from 8 pigs divided in 3 groups [68]

^c^ Mean of positivity rate estimated on pen-based oral fluids and individual buccal swabs (frothy saliva) collected from 15 pigs, sampling 3 rolling 5-pigs groups [10]

^d^ Mean of positivity rate estimated on pen-based oral fluids and individual buccal swabs collected from 12 pigs divided in 3 groups [62]

## How to collect oral fluids

Oral fluid sample collection is possible because it is consistent with pigs’ natural behavior. Pigs are curious and will readily explore unfamiliar objects, e.g., a rope dangling in the pen, by biting and chewing [28]. Pig are also highly social and if one member of the group interacts with the rope other pigs will follow [69, 70]. Thus, because it is voluntary and does not require animal restraint, oral fluid sampling is a welfare-friendly process with no discomfort or stress to pigs or animal caretakers [26]. Numerous videos illustrating the oral fluids collection process are available on the internet [71], but the following comments may be helpful.

### Oral fluid sampling basics

Oral fluid samples are readily collected from groups of pigs ≥21 days of age by providing access to a suspended length of cotton rope for ~ 30 min [64, 72, 73]. The majority of oral fluid research has been done on pens of ~ 25 pigs, in which case one rope will provide an oral fluid sample representing ~ 80% of the animals in a 30 min sampling [74, 75]. Samples are most easily collected in the morning when pigs first awake and prior to feeding, if on a feeding schedule [69, 75]. Allow 45 to 60 min of sampling time at the first collection for pigs to learn the process. Thereafter, the pigs will remember and will respond quickly to the presence of the hanging rope and 30 min should be sufficient to obtain the sample [15, 69, 73]. To harvest the sample, remove the rope, place the wet portion of the rope inside a plastic bag, extract the oral fluid (by hand or wringer), and decant the sample into a container [76]. Oral fluids can likewise be collected from individual animals, as is done in sows, gilts, or some boar studs. However, sampling individuals tends to be less successful than group sampling, particularly older sows and boars, although “training” the animals (see description in trouble-shooting section) can be helpful [15, 73, 75].

As opposed to nylon, hemp, or polyester, cotton rope provides the best overall diagnostic utility for antibody and nucleic acid detection [22, 39]. Three-stranded cotton rope ~ 1.6 cm in diameter is optimal for oral fluid collection in animals ≥40 kg, but younger animals prefer smaller diameter rope (≤ 0.8 cm). Alternatively, individual strands of larger diameter rope can be used to meet the younger pigs’ thinner rope preference [70, 73].

In the field, ropes are commonly suspended from pen gates, thereby avoiding the need to enter the pen either to hang or collect the rope. However, providing additional space around the rope will increase the number of pigs interacting with the rope at any one time [74, 76]. Strategies for providing additional space include hanging the rope from a rafter or from a bracket extending from a side of the pen [64, 77].

### Take care of the sample

The stability of antibody in swine oral fluids is poorly described and the data available is limited to PRRSV. Antibody degradation in oral fluids is temperature-dependent, e.g., PRRSV ELISA S/P (sample-to-positive) ratios in samples stored at 4 °C were stable for 12–14 days, with faster degradation coinciding with increasing temperature. No antibody was detected in oral fluid after 72 h at 30 °C [78, 79]. Freezing oral fluid samples for subsequent PRRSV antibody testing is a safe option, i.e., antibody oral fluid is highly resistant to “freeze-thaw” degradation, as indicated by consistent PRRSV ELISA S/*P* values in samples subjected to repeated “freeze-thaw” cycles (unpublished data).

Likewise, little quantitative information is available regarding the degradation of intact virus or viral nucleic acids in oral fluids. Calculations based on published data [79] estimated the half-life of RT-PCR-detectable PRRSV RNA in oral fluids as approximately 13 h (30 °C), 42 h (20 °C), and ≥ 14 days (< 10 °C). Unlike antibody, nucleic acids are susceptible to freeze-thaw degradation. This effect is undoubtedly not uniform across viruses, i.e., IAV RNA is particularly susceptible to this effect (unpublished data). To achieve the best results on frozen samples, Weiser et al. (2018) showed that thawing frozen oral fluids overnight at 4 °C produced more PRRSV RT-PCR positive results than thawing at 22 °C (94% vs 80% on matched samples, respectively) [80].

In summary, to maintain diagnostic targets (antibody and/or nucleic acids) when collecting oral fluids in the field, chill samples as soon as possible after collection by refrigeration (4 °C) or by placing the samples in coolers containing crushed ice or ice packs. The cold chain should be maintained throughout transport and in the laboratory until tested. If it is not possible to maintain the cold chain and/or testing cannot be completed within 7 days, samples should be frozen (≤ − 20 °C). However, multiple freeze-thaw cycles should be avoided, particularly in the case of samples intended for PCR testing. It follows that, long-term storage in self-defrosting freezers must be avoided because of temperature fluctuations during the defrost cycle. Attempts to stabilize oral fluids antibody using antimicrobials or oral fluids RNA using nucleic acid stabilizers showed no improvement when compared to chilling (4 °C) samples [79, 81, 82]. The use of Flinders Technology Associates (FTA) cards to preserve oral fluids PRRSV RNA was effective, but showed significant loss of RT-PCR sensitivity [83].

### Trouble-shooting oral fluid collection

**Fig. 2:**
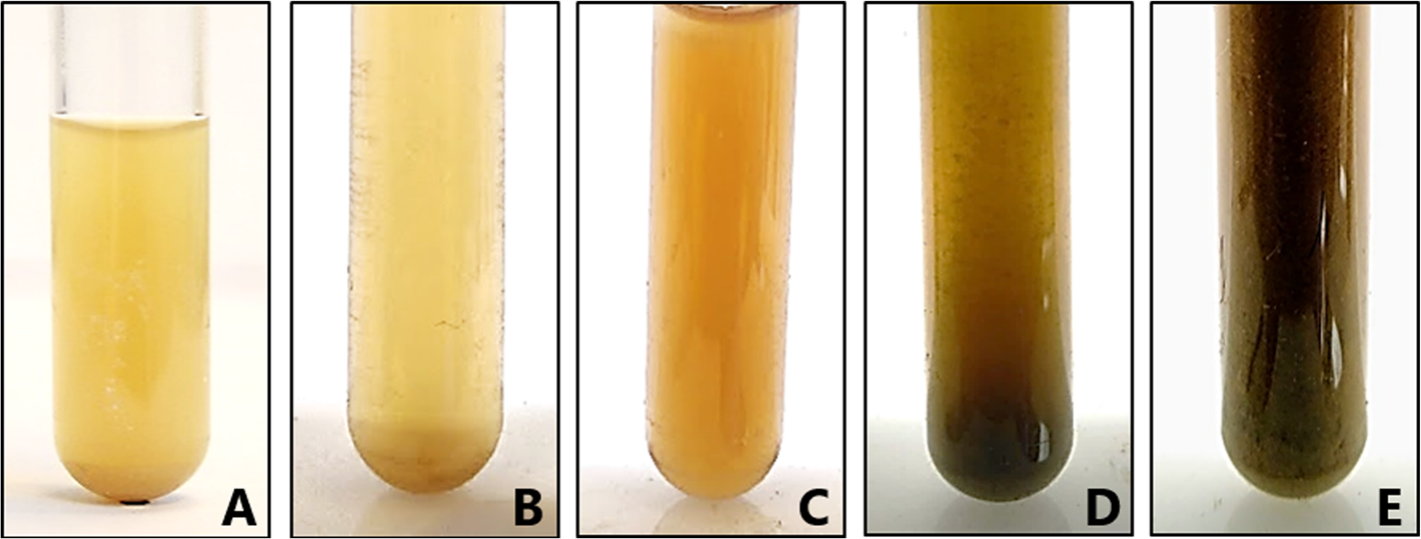
Appearance variability among swine oral fluid samples. **a** Oral fluids collected under experimental conditions. **b** Field oral fluids collected from individual-pen held pigs. **c**-**e** Field oral fluids collected from pen-held group of pigs

When collected under clean conditions, e.g., experimental settings, oral fluids are straw-colored and translucent. When collected under field conditions, oral fluids contain environmental contaminates, e.g., manure and feed, to varying degrees (Fig. 2a-e). Sample contamination may be reduced by not allowing the rope to reach the floor, i.e., set the bottom of the rope at pigs’ shoulder height [15, 74]. In the laboratory, organic contaminants per se do not affect the diagnostic properties of the sample (nucleic acid or antibody detection), but cleaner samples are easier to process and more amenable to accurate pipetting. Variable centrifugation protocols for swine oral fluids have been reported in literature, e.g., 12,000 g × 8 h [37], 14,000 g × 30 s [84], 9000 g × 10 min [64]. Gibert et al. (2017) reported that centrifuging at 15000 g × 15 min improved PRRSV nucleic acids detection in spiked samples compared to no centrifuged samples [85]. However, a “gentle” centrifugation protocol (3000 *g* × 5–10 min) should be helpful in eliminating large particles. Efforts to fully remove suspended particulates, e.g., chemical clarification [78], filtration [22], or prolonged centrifugation have shown no benefit in terms of improved diagnostic performance and may rise the sample processing time.

Some pigs, particularly younger pigs, may be reluctant to approach the hanging rope upon their first exposure. Enticing pig participation by providing rope with flavored (fruits juice, sucrose,) [86], colored, or aromatic substances (garlic, unpublished data) has generally not been rewarding with the exception of one report showing a higher oral fluid collection success rate in suckling piglets exposed to rope treated with a commercial baby pig supplement [70]. However, the initial reluctance to approach the rope can often be overcome by training the pigs, i.e., placing the rope on the floor for the pigs to investigate (20 min), slowly dragging the rope to the spot where it will be hung (the pigs will follow the “tail” of the rope), and then hanging a new clean rope [38, 74]. As is desirable for working with pigs, this process should be done quietly and without sudden movements so as to avoid startling the animals. Some individually housed adults may also be trained (as described above) to oral fluid collection [15, 75].

Little information is available on collecting oral fluids from pigs housed with organic bedding or under free range conditions, i.e., situations in which the presence of a hanging rope may not attract the pigs’ attention [75, 87]. In free range animals, oral fluids have been collected using “samplers” consisting of short lengths (~ 10 cm) of rope embedded with a cereal-based bait matrix. Investigators have reported that the animals chewed the samplers, dropped them on the floor, and the next pig repeated the process, thereby providing an “aggregate” specimen [44, 65]. Under experimental conditions, this approach provided for better CSFV or FMDV nucleic acids detection than oropharyngeal, nasal, or buccal swabs [44, 65, 88]. Several rope samplers can be provided to a group of pigs. Samplers should be recovered while still moist to maximize oral fluid collection.

## In the field

### Detection at the pen level

As the proportion of infected pigs in a pen increases, the probability of a positive test result (PCR or ELISA) likewise increases. Olsen et al. (2013b) quantified this relationship for PRRSV by establishing prevalence (0 to 36%) in pens of 25 pigs using MLV PRRSV vaccinated pigs (14 days prior). Oral fluids were then collected and tested for PRRSV RNA and antibody. For this review, data from Olsen et al. (2013b) were analyzed in a logistic regression model to establish the relationship between the positive oral fluid testing results (RNA or antibody) and within pen prevalence (Table 4). Data on detection by prevalence in pens of larger size is lacking. This is a concern because the industry is trending toward larger pens (up to 500 pigs in some systems). If the population of interest is a pen(s), current information would suggest using one rope per 25 pigs in order to optimize the proportion of pigs interacting with the ropes and, thereby, increase the probability of detection [74, 77, 87].

**Table 4:** Probability of PRRSV detection as a function of within pen prevalence

Within pen prevalence	Probability (%) of detection with one oral fluid sample (95% CI)^a^		Serum samples to match oral fluid probability^b^	
	RT-PCR	ELISA	RT-PCR	ELISA
5%	31% (9, 67)	17% (6, 38)	8	5
10%	79% (48, 94)	59% (37, 77)	11	5
20%	98% (88, 100)	94% (82, 98)	13	10
30%	100% (96, 100)	99% (93, 100)	12	10
40%	100% (98, 100)	100% (97, 100)	10	9
50%	100% (99, 100)	100% (98, 100)	9	8

^a^Probability of detection in oral fluids estimated by logistic regression (pen as random effect) from data reported in Olsen et al. (2013b). Within pen prevalence established by placing PRRSV-positive pigs (14 days after MLV vaccination) in pens of PRRSV-negative pigs to achieve 25 pigs per pen [89]

^b^Number of serum samples required to match the probability of detection for one oral fluid sample was estimated using a hypergeometric distribution

### Detection at the barn and site levels

**Fig. 3:**
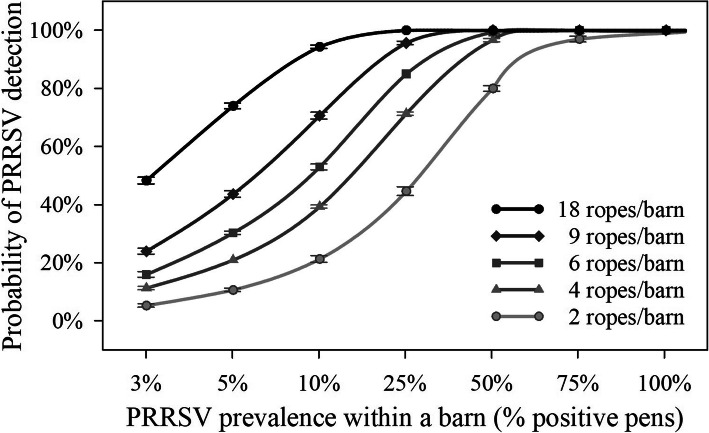
Probability of PRRSV detection within a barn (~ 1000 pigs) by pen prevalence and number of samples collected. Figure derived from data reported in Rotolo et al. (2017) [90]. Error bars represent the range of detection assuming assay diagnostic sensitivity of 95–100%

The surveillance objective in larger populations is determining the status of the barn or the production site (multiple barns). Rotolo et al. (2017) estimated the barn-level probability of PRRSV detection as a function of sample size, sampling location within the barn, and PRRSV prevalence (positive pens) [90]. Figure 3, derived from data in Rotolo et al. (2017), provides guidelines regarding the association between prevalence (number of positive pens) and the number of oral fluid samples required to detect PRRSV in the barn population. For example, collecting 6 pen-based oral fluids in a barn with ~ 1000 pigs would provide for a ~ 50% probability of PRRSV detection when 10% of pens are positive. However, this probability increases to ~ 80% when the number of positive pens increases to 25%. The original estimates were based on detection of RNA, but similar estimates would be expected for antibody. Likewise, a similar association would be expected for other viral pathogens.

When collected from a group of pigs, the aggregate and undiluted oral fluid samples represent such group of animals. Further pooling oral fluid samples collected within barns or on a production site is not recommended because of the potential for creating false negatives by diluting low concentrations of nucleic acids or antibody below the assay limit of detection, as is known to occur in serum [91]. Given a limited budget for surveillance, collecting fewer samples and establish their status with confidence is preferable to collecting a higher number of samples and pooling them.

Regardless of the number of samples collected, Rotolo et al., (2017) showed that fixed spatial sampling, i.e., spacing sampling points equidistant over the length of the barn, provided a higher probability of detection than random sampling. This reflects the spatially-dependent pattern of disease spread. That is, pathogens move from pig-to-pig and pen-to-pen; they are not randomly distributed within a barn [90].

In commercial systems, on-going site-level surveillance based on collecting a limited number of samples from all barns at fixed time intervals is preferable to collecting many samples sporadically [90]. A systematic approach provides real-time information on the dynamics of viral spread. For example, Ramirez et al. (2012) collected 6 oral fluids every 2 weeks from the same pens in 10 wean-to-finish barns on 10 different production sites for 18 weeks (*n* = 600 oral fluid samples, representing 12,150 pigs). Nucleic acid testing for PCV2, torque teno virus (TTV)1, TTV2, IAV, and PRRSV showed the presence of various combinations of viral infections on a continuous basis in all barns; albeit, the timing of infections was highly variable, even among barns in the same production system [64].

## Be an intelligent consumer

Testing for surveillance is fundamentally different from testing to achieve a diagnosis. That is, diagnostic assays typically use cutoffs that find a balance between diagnostic sensitivity and diagnostic specificity (Youden Index). In contrast, surveillance assays must provide near-perfect diagnostic specificity (no false positives!) even at the cost of lower diagnostic sensitivity. This because false alarms trigger disruption in production and quickly poison the consumer’s confidence in the surveillance system. In some cases, improvement in diagnostic specificity can be achieved with minimal impact on diagnostic sensitivity [92]. For example, in an evaluation of a PRRSV oral fluid ELISA (*n* = 2205 oral fluid samples), Henao-Diaz et al. (2020) found that changing the ELISA’s cutoff from S/*P* ≥ 0.4 to S/*P* ≥ 1.0 lowered diagnostic sensitivity from 99.7 to 96.2%, but raised diagnostic specificity from 98.1 to 100% [93]. Pragmatically, this lowered diagnostic sensitivity represented a < 3 day delay in detection as the infected animals’ antibody response reached S/*P* ≥ 1.0. In the field, lower test sensitivity can be offset either by collecting more samples at each sampling or by using routine surveillance testing (every 2–4 weeks).

**Fig. 4:**
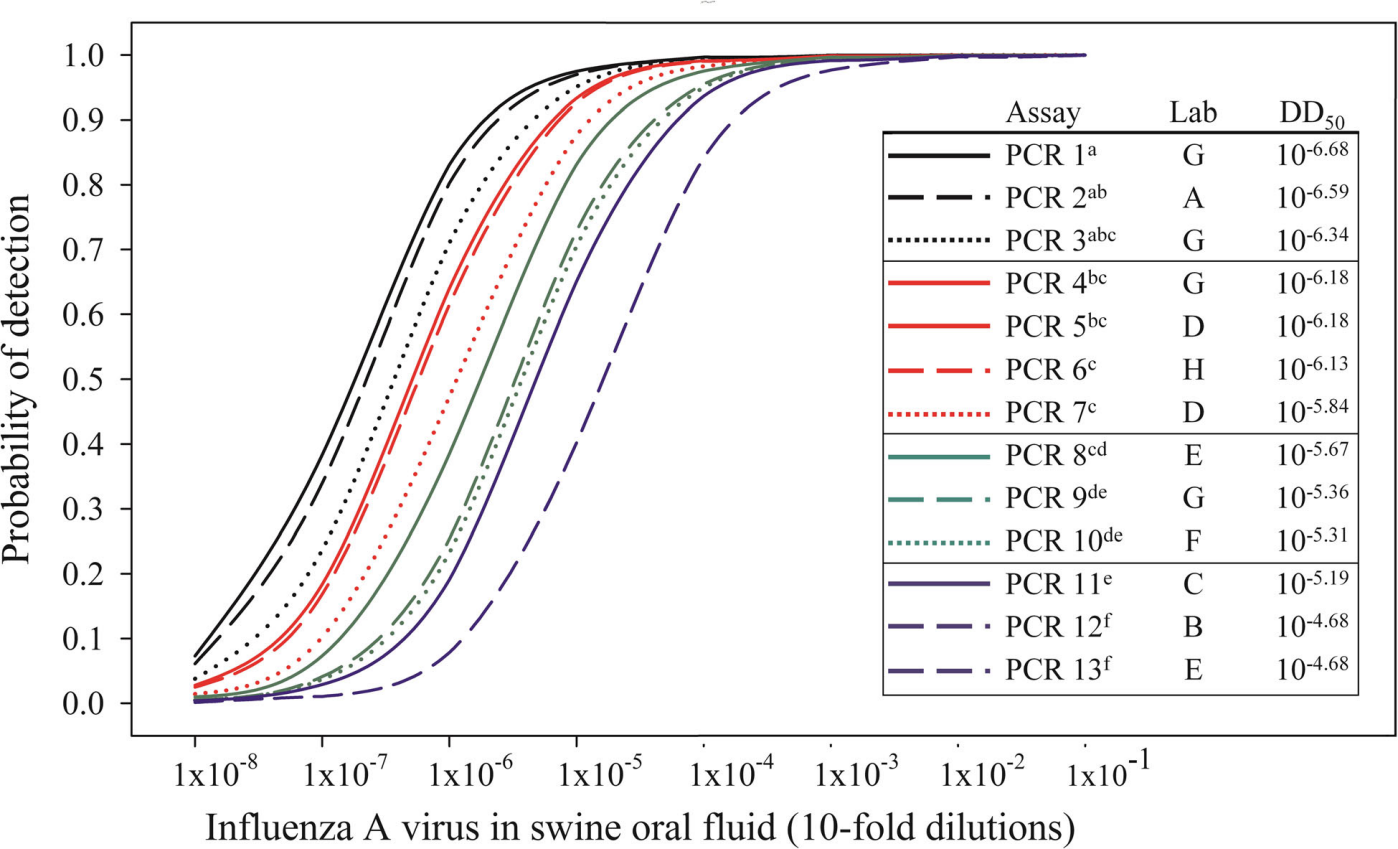
Probability of detecting influenza A virus (IAV) in swine oral fluids by RT-qPCR. Superscripts reflect significant differences (*p* < 0.05) in assay performance between 13 assays performed in 8 laboratories (A to H). Row groups indicate similar probability detection dose 50 (DD_50_), that is, the inoculum dilution at which there was a 50% probability of detection (10^–4.68^ to 10^–6.68^). Reprinted from Goodell et al. (2016) [24] Canadian Journal of Veterinary Research 80, 12–20 (copyright 2016) with permission (#P2020–0011)

There are no perfect tests, but some tests are better than others. Thus, marked differences in diagnostic performance were reported among commercial PRRSV oral fluid antibody ELISAs and among IAV oral fluid RT-PCRs [24, 38] (Fig. 4). Since testing cannot be perfect, the use of statistical process control charting can be used provide a historical context for interpreting test results. In addition, have a plan in place for dealing with unexpected results. The plan may include retesting the original sample (in duplicate) and/or resampling the population. A robust confirmatory testing strategy can combine the strengths of both antibody and nucleic acid detection [66].

## Conclusions

From an animal health perspective, larger pig populations combined with the extensive movement of pigs, people, and material between production sites produce conditions that promote the circulation of infectious diseases within/between farms [4, 5]. These circumstances challenge our ability to prevent, control, and/or eliminate impactful swine pathogens. Disease management based on clinical observations is neither timely nor accurate; we require an active and systematic process that achieves the timely detection of pathogens and produces useful data that can guide management decisions. The last decade has seen significant advances in the routine use of aggregate specimens in surveillance, including oral fluids. Further improvements may be anticipated as additional diagnostic assays specifically adapted to the oral fluid matrix emerge and statistical refinements in the application of oral fluid sampling to populations are achieved.

## Data Availability

Not applicable.

## Abbreviations

APPV: Atypical porcine pestivirus
ASFV: African swine fever virus.
CSFV: Classical swine fever virus
Diagnostic sensitivity: Probability that a positive test result comes from an infected pig
Diagnostic specificity: Probability that a negative test result comes from an uninfected pig
ELISA: Enzyme-linked immunosorbent assay
FMDV: Foot and mouth disease virus
IAV: Swine influenza A virus
HEV: Hepatitis E virus
Ig: Immunoglobulins
JEV: Japanese encephalitis virus
MLV: Modified live vaccine
PCR: Polymerase chain reaction
PCV: Porcine circovirus
PDCoV: Porcine delta coronavirus
PEDV: Porcine epidemic diarrhea virus
PHEV: Porcine hemagglutinating encephalomyelitis virus
PKV: Porcine kobuvirus
PPIV1: Porcine parainfluenza virus 1
PPV: Porcine parvovirus
PRCV: Porcine respiratory coronavirus
PRRSV: Porcine reproductive and respiratory virus
PRV: Pseudorabies virus (Aujeszky’s)
qPCR: Quantitative polymerase chain reaction
RT-PCR: Reverse transcription polymerase chain reaction
RT-qPCR or qRT-PCR: Reverse transcription quantitative polymerase chain reaction
SVA: Senecavirus A
SVDV: Swine vesicular disease virus
TGEV: Transmissible gastroenteritis virus
TTV: Torque teno virus
